# Pain, quality of life, and integral management in a cohort of patients diagnosed with hypophosphatasia in Colombia

**DOI:** 10.1186/s13023-024-03366-9

**Published:** 2024-11-06

**Authors:** Jorge Armando Rojas Martínez, Ana María Zarante Bahamón, Luz Victoria Salazar, Andrés Felipe Morales, María Fernanda Higuera Cristancho, Juliana Villanueva Congote, Ignacio Zarante Montoya, Lina María Gómez Espitia

**Affiliations:** 1 Asociación Colombiana de Médicos Genetistas (ACMGen), Cra. 7 #40-62 edificio 32, Chapinero, Bogotá D.C., Cundinamarca, Colombia; 2 Asociación Colombiana de Pacientes con Enfermedades de Depósito Lisosomal (ACOPEL), Bogotá D.C., Colombia; 3 Pontificia Universidad Javeriana, Bogotá D.C., Colombia; 4 NeuroEconomix, Bogotá D.C., Colombia

**Keywords:** Health access, Asfotase alfa, Quality of life, Pain, Hypophosphatasia, Hypomineralization, PRO

## Abstract

**Background:**

Hypophosphatasia (HPP; OMIM 241510, 241500, and 146300) is a progressive metabolic, genetic disease with wide clinical heterogeneity, ranging from perinatal lethality to mild or moderate localized symptoms. This study aims to analyze the perception of pain, quality of life, and access barriers to healthcare among patients diagnosed with hypophosphatasia in Colombia. In this document we present pain and quality of life results.

**Methods:**

This study is an observational cohort of 18 HPP patients registered in the Colombian Association of Patients with Lysosomal Storage Diseases and Other Orphan Diseases (ACOPEL) database. We conducted a descriptive analysis using data from three questionnaires (SF-36, Brief Pain Questionnaire (BPQ), and Hypophosphatasia Impact Patient Survey (HIPS); the latter was translated into Spanish and validated for this study.

**Results:**

The most affected features, according to the SF-36 questionnaire, were overall health, vitality, and pain, with a median score above 67%. Patients' perception of their health status (HIPS questionnaire) was favorable, with 38.9% of cases reporting excellent health. On average, results from the BPQ indicated mild to moderate intensity of current pain experienced by patients. Consistency was observed in the reports of either the absence of pain or the presence of mild to moderate intensity when analyzing the results of the three questionnaires.

**Conclusions:**

Colombian patients with HPP experience mild to moderate impairment in quality of life and pain that interfere with their daily activities. However, there is wide variability in the results obtained.

## Introduction

Hypophosphatasia (HPP; OMIM 241510, 241,500, and 146,300) is a progressive metabolic genetic disease caused by mutations in the *ALPL* gene, which encodes the non-tissue-specific alkaline phosphatase protein [1–3]. Its deficiency leads to various health issues, including diminished bone mineralization, dental problems, seizures, persistent pain, and muscle weakness [2, 4–6].

The clinical presentation of HPP is highly diverse, ranging from perinatal lethality to individuals experiencing localized symptoms of mild to moderate severity [4, 7]. The phenotypic variability, added to the low prevalence, makes diagnosing HPP a significant challenge [3]. Diagnosis is usually based on a clinical history of musculoskeletal manifestations associated with persistent low serum alkaline phosphatase levels for age and sex [8]. However, sequencing or study of deletions and duplications of the *ALPL* gene is an optional criterion [9].

Currently, no curative treatments are available, and management is addressed to impact patients' quality of life [10]. Enzyme replacement therapy (ERT) has demonstrated its ability to enhance these patients' quality of life and survival by modifying the disease's natural course [11, 12].

Because hypophosphatasia is an orphan disease, it is frequently evaluated using case reports, single-center studies, and retrospective reviews with few patients. Indeed, few studies have been published in Latin America, and, to our knowledge, no similar article has been published on this disease in the country; given its low prevalence, patient recruitment is difficult.

Internationally, the prevalence of severe forms has been reported to range from 1 in 100,000 to 450,000 births, depending on the reporting country [13–15]. In Colombia, the prevalence of this condition is not known with accuracy and it is believed that there may be an underdiagnosis.

This study aims to analyze the perception of pain and quality of life in patients diagnosed with hypophosphatasia in Colombia while evaluating the impact of ERT, providing an overview of the patients within the country.

## Methods

This study is a descriptive observational cohort of patients with hypophosphatasia, confirmed by clinical manifestations and biochemical studies. All patients in the study underwent molecular analysis as part of their disease evaluation. We selected participants from the Colombian Association of Patients with Lysosomal Storage Disorders and Other Rare Diseases database (ACOPEL), and they were also included in the patients who belonged to the Colombian National Registry of Rare Diseases.

Socio-demographic characteristics of the patients were analyzed using frequency distribution. ERT was also explored at the time of the study. All patients were administered the SF-36, the brief pain questionnaire (BPQ), and the Hypophosphatasia Impact Patient Survey (HIPS). A trained psychologist conducted the surveys via telephone, video calls, or in-person sessions. The scores of the three scales of their domains are presented descriptively, without association or hypothesis testing. The data distribution was evaluated using means and standard deviations (SD) or median and interquartile range for quantitative variables. For qualitative variables, the absolute and relative frequency was reported. The symptom duration for each patient was estimated by calculating the time elapsed from the age of symptom onset to the time of inclusion in the study.

The HIPS questionnaire was validated only in appearance for use in this study by following these steps. After receiving permission from Alexion Inc (the questionnaire creator), we translated the questionnaire directly from English to Spanish. The Spanish version was reviewed by two geneticists, two members of ACOPEL, and an interdisciplinary team consisting of neurologists, epidemiologists, and economists. All comments from the reviewers were incorporated into the questionnaire. Subsequently, we consulted two clinical experts to review one specific question in the Spanish version, validate adjustments made for the Colombian context, and ensure the overall clarity of all questions.

The study protocol was approved by the Institutional Research and Ethics Committee of the medical school of the Pontificia Universidad Javeriana and the Hospital Universitario San Ignacio (ID number 2022/078). Before starting the study, all patients signed the informed consent form. Patients under 18 had to have a guardian's informed consent signed and agreed to participate in the study. The study took approximately six months to complete, beginning after ethics committee approval in May 2022. Data collection took place in July and August, and data analysis took place in September and October.

## Results

Eighteen patients participated in the study, ranging in age from 3 to 33 years, with a mean age of 13.8 (SD 8.4 years) (Table 1); five patients were under eight (27.8%), eight patients were between eight and sixteen (44.4%), and five were 18 or older (27.8%) (Table 1). Gender distribution showed seven females (38.9%) and 11 males (61.1%). Health insurance types included 8 with private coverage (44.4%) and 10 with public insurance (55.6%). Patients hailed from various Colombian regions, with Bogotá being the most represented (22.22%) (Table 1); all patients speak Spanish and none have a special ethnicity. All patients were classified with pediatric-onset hypophosphatasia based on the age of the first disease signs and symptoms.

**Table 1:** Characteristics of the patients included in the study

Characteristics	Mean (SD)			Median (IQR)		
	Female n = 7	Male n = 11	Total n = 18	Female n = 7	Male n = 11	Total n = 18
Age (years)	13.6 (10.1)	13.9 (8.3)	13.8 (8.7)	12 (7–17)	15 (8–18)	13.5 (6–18)
Height (cms)	122 (24.2)	135 (27.3)	130 (26.3)	119 (104–145)	145 (111–158)	134 (110–147)
Weight (kg)	29.1 (14.8)	38.9 (21.6)	35.1 (19.4)	24 (16–40)	34 (20–60)	30.5 (18–50)

Characteristics	Female		Male		Total	
	n	%	n	%	n	%
Health insurance						
Contributive	2	28.6	6	54.5	8	44.4
Subsidized	5	71.4	5	45.5	10	55.6
Residence Department						
Atlántico	2	28.6%	1	9.1	3	16.67
Bogotá†	0	0.0%	4	36.4	4	22.22
Boyacá	0	0.0%	1	9.1	1	5.56
Cesar	0	0.0%	2	18.2	2	11.11
Cundinamarca	2	28.6%	1	9.1	3	16.67
Córdoba	1	14.3%	0	0.0	1	5.56
Santander	2	28.6%	1	9.1	3	16.67
Valle del Cauca†	0	0.0%	1	9.1	1	5.56
Lives with*						
Mother/stepmother	4	26.7	10	34.5	14	77.8
Father/stepfather	4	26.7	9	31.0	13	72.2
Grandmother/Grandfather	3	20.0	1	3.4	4	22.2
Siblings	2	13.3	9	31.0	11	61.1
Son	1	6.7	0	0.0	1	5.6
Partner	1	6.7	0	0.0	1	5.6

SD: standard desviation. IQR: interquartile range

^†^One of the patients living in Bogotá was born in Caquetá, and the patient living in Valle del Cauca was born in Florida, USA. For all other patients, the resident department is the same as the place of birth

^*^For this variable, the percentages do not add up to 100% as the patient could respond to more than one category

### Molecular study and ERT

Although in Colombia, the genetic study is not mandatory to confirm and report the disease, the 18 patients had a genetic study performed by different methodologies (Sanger sequencing, NGS and qPCR of the *ALPL* gene and NGS panel for skeletal dysplasias), in which at least one variant in the *ALPL* gene was evidenced, most of them being autosomal recessive forms (Table 2). The most frequent allele in the patients was c.892G > A p.(Glu298Lys), always associated with another variant in trans. Missense variants were the most frequent; a small duplication detected by sequencing was documented. No major rearrangements in the gene were reported. However, most heterozygous patients did not provide additional studies to rule out the presence of another variant.

**Table 2:** Information about the molecular result, age at the time of molecular study of hypophosphatasia, and time with the disease

Patient	Allele 1	In Silico Prediction and Allelic Frequency – Allele 1	Allele 2	Age at the time of the study (years)	Age at molecular study	Time with HPP^±^ (years)	Age of symptom onset	Time from symptom onset to molecular study*
1	c.892G > A, p.(Glu298Lys	PolyPhen: Probably pathogenic Align-GVGD: C0 SIFT: Tolerated MutationTaster: Pathogenic Conservation: moderate nt/moderate nt/moderate aa gnomAD: 0.00003 TOPMed: 0.00001 ExAC: 0.00002	c.892G > A, p.(Glu298Lys)	22	14 years	7.9	One year	13 years
2	c.892G > A, p.(Glu298Lys	–	c.892G > A,p.(Glu298Lys)	16	10 years	6.0	10 years	0
3	c.455G > A,p.(Arg152His)	PolyPhen: Benign SIFT: Tolerated MutationTaster: Benign Conservation: Benign (Supporting) gnomAD: 0.00814 TOPMed: 0.00791 ExAC: 0.01145	–	15	9 years	6.1	8 years	1 year
4	c.571G > A,p.(Glu191Lys)	PolyPhen: Uncertain Align-GVGD: C0 SIFT: Uncertain MutationTaster: Pathogenic Conservation: moderate nt/moderate nt/moderate aa gnomAD: 0.00200 TOPMed: 0.00065 ExAC: 0.00258	c.343_348dup (p.Thr115_Ala116dup)§	16	11 years	5.4	10 years	1 year
5	c.659G > C, p.(Gly220Ala)	PolyPhen: Pathogenic Align-GVGD: C0 SIFT: Uncertain MutationTaster: Pathogenic Conservation: moderate nt/moderate nt/moderate aa gnomAD:not found TOPMed: not found ExAC not found	c.659G > C, p.(Gly220Ala)	18	12 years	5.8	4 years	8 years
6	c.892G > A p.(Glu298Lys)	–	c.892G > A p.(Glu298Lys)	20	14 years	5.9	2 years	12 years
7	c.382G > A p.(Val128Met)	PolyPhen: Probably pathogenic Align-GVGD: C0 SIFT:Uncertain MutationTaster: Pathogenic Conservation: moderate nt/moderate nt/moderate aa gnomAD: not found TOPMed: not found ExAC: not found	c.382G > A p.(Val128Met)	6	1 year and 4 months	4.6	1 year	4 months
8	c.455G > A p.(Arg152His)	–	–	10	5 years	4.6	3 years	2 years
9	c.382G > A p.(Val128Met)	–	–	6	2 years	4.0	1 year	1 year
10	c.659G > C p.(Gly220Ala)	–	c.659G > C p.(Gly220Ala)	4	1 month	3.9	1 year	0‡
11	c.394G > A p.(ala132Thr)	PolyPhen: Probably pathogenic Align-GVGD: C0 SIFT:Uncertain MutationTaster: Pathogenic Conservation: moderate nt/moderate nt/moderate aa gnomAD: not found TOPMed: not found ExAC: 0.00000398	571G > A p.(Glu191Lys)	31	26 years	4.5	1 year	25 years
12	c.485G > A p.(Gly162Asp)	PolyPhen: Probably pathogenic Align-GVGD: C0 SIFT: Deleterious MutationTaster: Pathogenic Conservation: moderate nt/moderate nt/moderate aa gnomAD: not found TOPMed: not found ExAC: not found	–	12	7 years	4.6	6 years	1 year
13	c.659G > C p.(Gly220Ala)	–	c.659G > C p.(Gly220Ala)	34	30 years	4.3	3 years	27 years
14	c.892G > A p.(Glu298Lys)	–	c.892G > A p.(Glu298Lys)	4	1 month	3.9	Less than 1 year	0‡
15	c.334G > C p.(Gly112Arg)	PolyPhen: Probably pathogenic Align-GVGD: C0 SIFT: Deleterious MutationTaster: Pathogenic Conservation: moderate nt/moderate nt/moderate aa gnomAD: not found TOPMed: not found ExAC: 0.00000398	–	16	13 years	2.8	2 years	11 years
16	c.571G > A p.(Glu191Lys)	–	–	8	5 years	3.0	4 years	1 year
17	c.382G > A p.(Val128Met)	–	c.382G > A p.(Val128Met)	3	10 months	2.2	1 year	0‡
18	c.457 T > A p.(Trp153Arg)	PolyPhen: Probably pathogenic Align-GVGD: C0 SIFT: Benign MutationTaster: Pathogenic	–	10	7 years	3.2	7 years	0

Source: database of patients linked to ACOPEL as of the 31 of December 2022. ^±^ Time with HPP was calculated from age at the time of the study and age at molecular testing. *The time was calculated from the age at molecular testing and the age of symptom onset; this time is approximate since the exact date of symptom onset is unknown. ‡ These patients have an age of symptom onset that is older than the age at molecular testing and was therefore considered to be less than 1 year. § In Silico Prediction and Allelic Frequency – Allele 2 Patient 4: ACMG Classification: Likely Pathogenic, Conservation Scores phyloP100: 9.619, gnomAD: not found, TOPMed: not found, ExAC: not found

Currently, c.485G > A and c.457T > A are classified as uncertain significance variants. The patient with variant c.455G > A, p.(Arg152His) in heterozygosis underwent a qPCR study, which was negative for deletions or duplications in the *ALPL* gene. Distribution of genotypes: 10 patients were homozygous (55.6%), six heterozygous (33.3%) and 2 compound heterozygous (11.1%).

The analysis of age at the time of the molecular study showed that seven patients were taken to molecular evaluation after ten years of age (38.9%), six patients were evaluated between 5 and 10 years of age (33.3%) and, finally, five patients underwent molecular testing between the first month of life and two years of age (27.8%). No patients were tested between 3 and 4 years of age. The median age in the molecular study was eight years (IQR between 3 and 13 years of age), ranging from 1 month to 30 years in the patients analyzed. The patients experienced this health condition for a duration spanning from 2 to 8 years (Table 2). This period was determined by calculating the time elapsed from the patient's age at the start of the molecular study to the time of the study itself. The date of the molecular study was selected as a reference point due to its objective clinical relevance for all patients. Notably, the precise date of diagnosis from the clinical history was not accessible. These data were obtained from the date of issue of the genetic result of the molecular study, information collected by the ACOPEL team.

The time elapsed between symptom onset and the time at molecular testing disclosed that, of the 18 patients, 11 (61.1%) were molecularly assessed within the first year after symptom onset, one patient was evaluated two years after symptom onset, and another at eight years, and five patients (27.8%) were assessed at least 11 years after symptom onset (Table 2).

In terms of allele frequencies, a total of 11 variants were identified. The allele distribution was as follows: c.892G > A 22%, c.659G > C 16.7%, c.382G > A 13.9%, c.571G > A 8.3%, and c.455G > A 5.6%. The alleles c.485G > A, c.457G > A, c.394G > A, c.343_348dup, and c.334G > C each appeared once (2.8%).

Concerning the specific treatment for the disease, ERT was administered to 94.4% of the patients at some stage, with 77.8% currently receiving it. The median age at treatment initiation was ten years, with varying initiation ages: 17.6% started at one year of age, 11.8% between 1.5 and 2 years, 23.5% between 5 and 10 years, and 47.1% between 11 and 30 years. Among those who received ERT, 16 out of 17 patients reported discontinuation, which occurred over a period ranging from 15 days to one year. Furthermore, 15 of 17 treated patients reported improvement upon starting the medication, especially in appetite, weight, skeletal health, gait, and pain reduction.

### SF-36 questionnaire results

Ten patients completed the questionnaires (55.6%) and, in eight cases, by the parent/guardian or caregiver (44.4%).

**Fig. 1:**
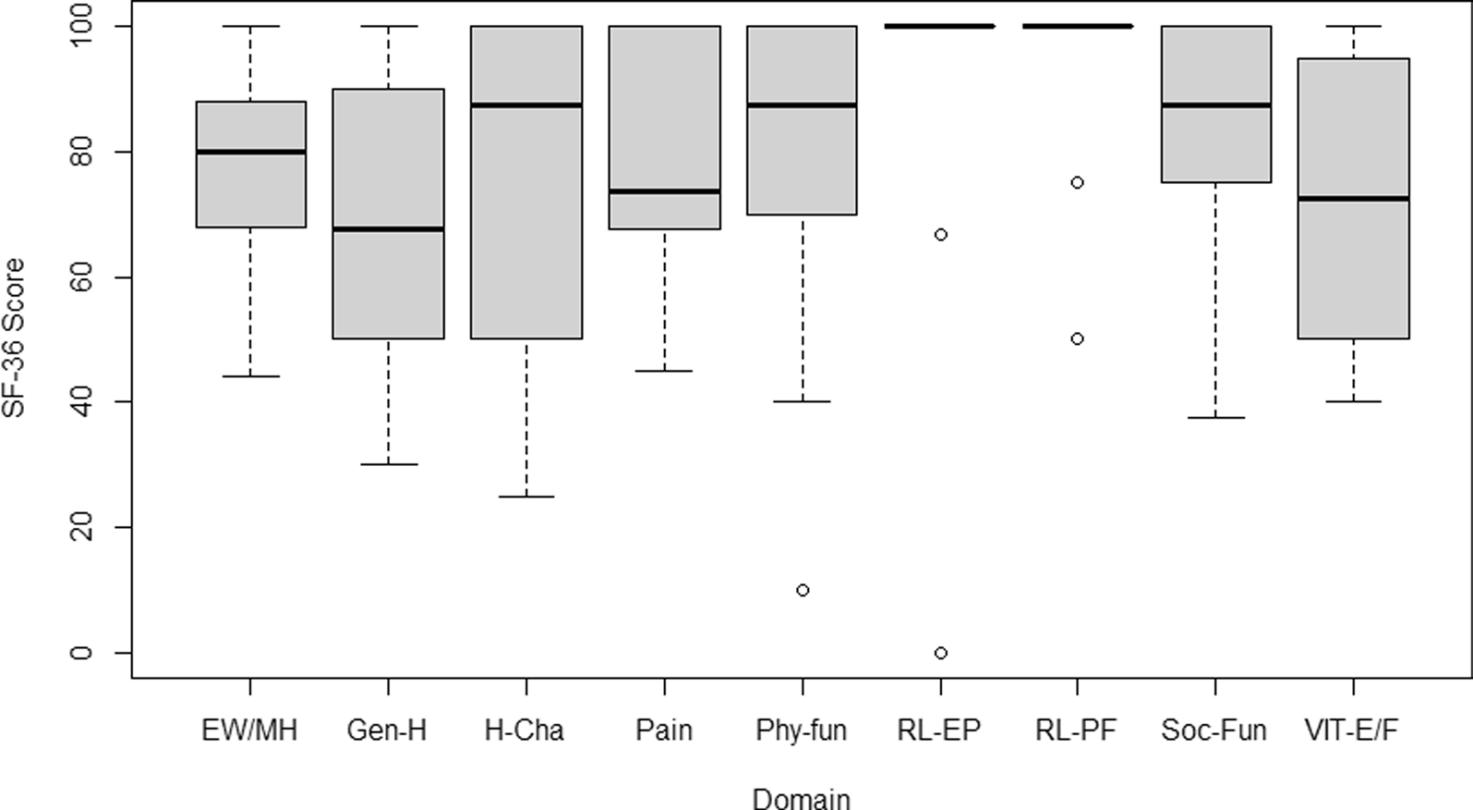
Results of the SF-36 questionnaire, according to the concept assessed. Source: own elaboration in RStudio®. EW/MH: Emotional well-being/mental health; Gen-H: General health; H-Cha: Health change or transition; Phy-fun: Physical functioning; RL-EP: Role limitations due to emotional problems; RL-PF: Role limitations due to physical health, Soc-Fun: Social functioning; VIT-E/F: Vitality: Energy/fatigue

The most affected aspects were general health, vitality, and pain, with a median score of 67.5% (IQR of 50% to 90%), 72.5% (IQR of 50% to 95%), and 73.8% (IQR of 67.5% to 100%), respectively. Likewise, the aspects that showed minor impairment in this cohort of HPP patients were role limitations due to physical health and emotional problems, with median scores of 100% in both areas. However, a minimum of 50% and 0% score was evident, respectively. Concerning physical functioning, role limitations due to physical health, and emotional problems, patients reported an acceptable health status (scores of 70% or higher). Nonetheless, a significant impairment was evident in some of the patients (Fig. 1).

### Brief pain questionnaire (BPQ) results

Based on the body diagram, 61.1% of patients reported pain, with varying levels of distribution: 54.5% in 2 to 3 body areas, 27.3% in four to six, and 9.1% in 10. In total, 45 different body sites reported pain, with the lower back (26.7%) and legs (15.6%) being the most affected areas. Elbows, knees, and thorax accounted for 8.8% of reported pain.

**Fig. 2:**
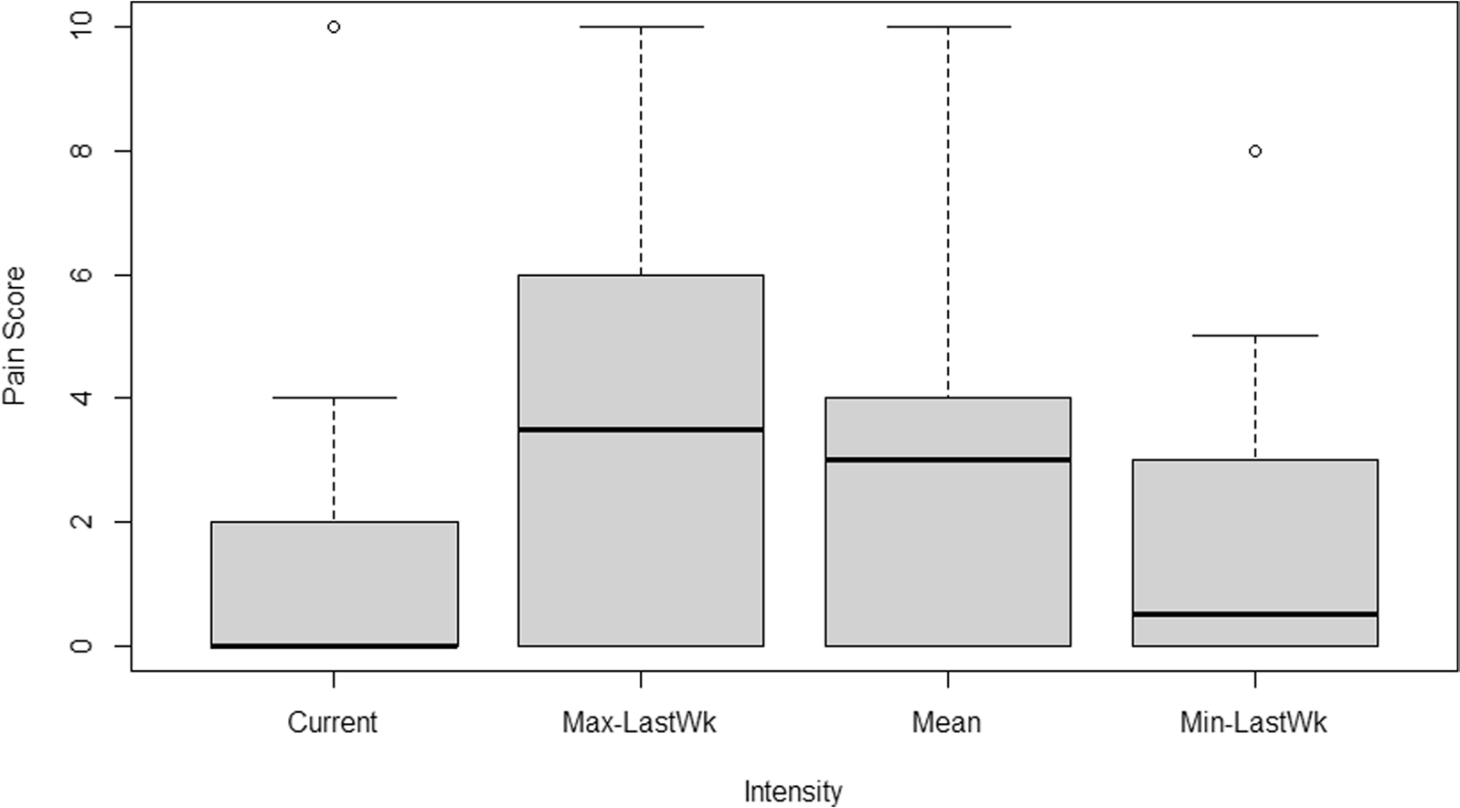
BPQ pain intensity results Source: own elaboration in RStudio®. Max-LastWk: Maximum in the last week; Min-LastWk: Minimum in the last week

The median scores of the maximum and minimum pain intensities experienced by patients in the past week were 3.5 and 0.5, with IQRs of 0 to 6 and 0 to 3, respectively. Furthermore, the median reported pain intensity was 3, with an IQR of 0 to 4. Current pain intensity displayed a median score of 0, with an IQR of 0 to 2, indicating that most patients experienced either no or mild pain during the study period (Fig. 2).

The pain severity, measured with a median score of 2.8 (IQR: 0.3–4), peaked at 5 points. Patients commonly employed stretching, analgesics, lying down, and walking as pain-relief strategies. Conversely, standing and lifting heavy objects were identified as pain-aggravating factors.

Five patients (27.8%) reported taking analgesic medication when analyzing pain treatment, and all reported receiving acetaminophen. The perception of pain relief generated by the analgesic treatments or medicines received during the last week demonstrated that of the five patients receiving this type of medicine; three reported a 100% improvement in pain during the previous week, one reported a 50% improvement, and another 40% improvement during the same period.

During the assessment of the primary cause of pain, two patients (11.1%) attributed their pain to the effects of treatment, which encompasses medication, surgery, radiation, and prostheses, as outlined in the questionnaire. Notably, these two patients were undergoing ERT (Asfotase alfa) treatment at the time of the study. On the other hand, eleven patients (61.1%) attribute their pain to the underlying disease itself, while five patients (27.8%) perceive their pain as unrelated to the primary pathology.

The adjective used by the patients to describe the type of pain presented showed that half of the patients reported their pain to be exhausting, and the adjectives reported by 30 to 45% of the patients were fatiguing (heavy), persistent, aching/continuous, and stabbing.

**Fig. 3:**
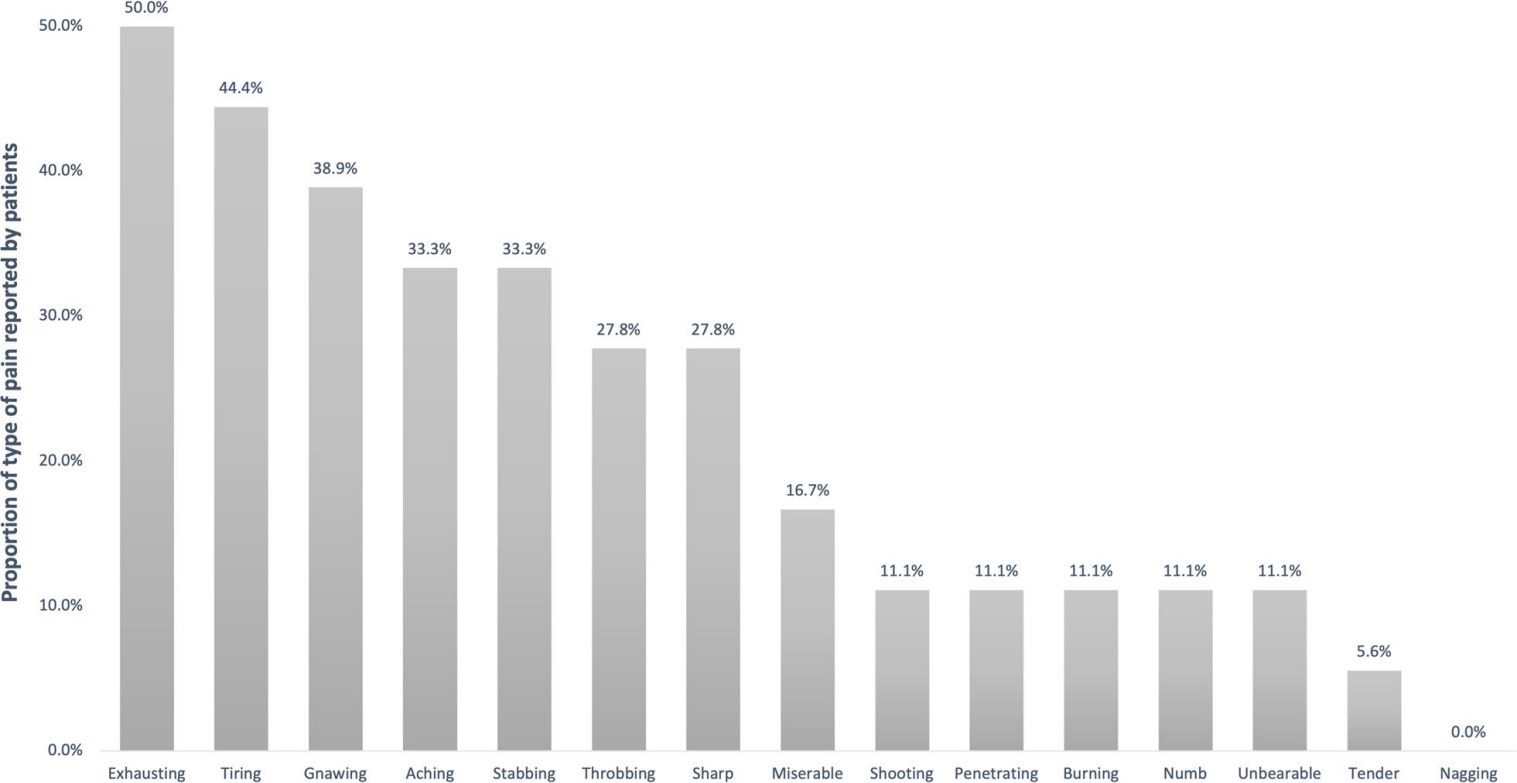
Results of the impact of pain on aspects of life during the last week. Source: own elaboration in Microsoft Excel®

Results about pain's impact on patient's lives in the past week reveal minimal interference with most activities, indicated by a median score of zero and an IQR from 0 to 3. However, mood disruption due to pain displayed more variability, with a median score of 1 and an IQR from 0 to 6 (Fig. 3).

When assessing pain interference, the median score was 1.5 (IQR: 0–3.3), with a maximum of 5.3 points. Regarding pain medication frequency, most (88.9% or 16 patients) did not take medication daily, while only two patients (11.1%) used it 1 to 2 times within 24 h.

Finally, when analyzing the need for stronger treatment or higher doses, most (83.3% and 94.4%) of patients did not feel it necessary. None of the patients reported experiencing pain medication side effects. In terms of alternative pain relief methods, 50% didn't use any, and 38.9% employed various methods, including massage (five patients), electrodes (one patient), and video games (one patient).

### HIPS results

In respect of the perception of health, the study revealed a median response of 2.5, representing a range from excellent to fair, with an IQR spanning from 1 to 4. It is worth highlighting that all patients reported their health as good, with a noteworthy 38.9% rating as excellent.

Considering limitations in the past four weeks in activities requiring energy or involving bending, standing, or stooping due to health issues, a median response of 4 (indicating no limitation) was reported for each activity. The IQR ranged from 2 (somewhat limited) to 4 (no limitation) for these specific activities. Furthermore, more than 55% of the patients responded that they had no limitations in performing these activities in both questions.

When evaluating the limitation to perform school-type activities/work or activities with friends due to physical health problems or because of emotional or behavioral issues, a median score of 4- with no limitation was found (IQR from 3- somewhat limited to 4- with no limitation and from 4 to 4, respectively). For these two questions, 72.2% and 83.3% of patients were found to have no limitations.

For pain or bodily discomfort assessment during the last four weeks, patients had a median score of 1.5 (IQR 1 to 3; none to mild); 50% had no pain, and none reported severe or severe pain. On the perception of satisfaction in the last four weeks with relationships with friends and with their life relationship in general, patients had a median score of 1 and 1.5, respectively (IQR 1 to 2; very satisfied to somewhat satisfied). For these two questions, 66.7% and 50% of patients were found to be very satisfied, and no patients were found to be very dissatisfied.

Concerning the behavior compared to people of the same age, the patients analyzed presented a median score of 1-excellent (IQR from 1 to 3; excellent to sound). It was identified that 55.6% and 33.3% of patients answered these questions as excellent and good, respectively. No patient reported poor behavior. On aspects related to family history, five patients reported having a family member with hypophosphatasia (27.8%).

The onset of hypophosphatasia symptoms typically appeared at a median age of 2.5 years, ranging from 1 to 6 years (minimum less than one year, maximum ten years). Most patients (72.2%) experienced symptoms within the first four years of life, while the remaining 27.8% reported symptom onset between 6 and 10 years of age. All patients developed symptoms before turning 18, categorizing them as pediatric HPP cases in this study.

**Fig. 4:**
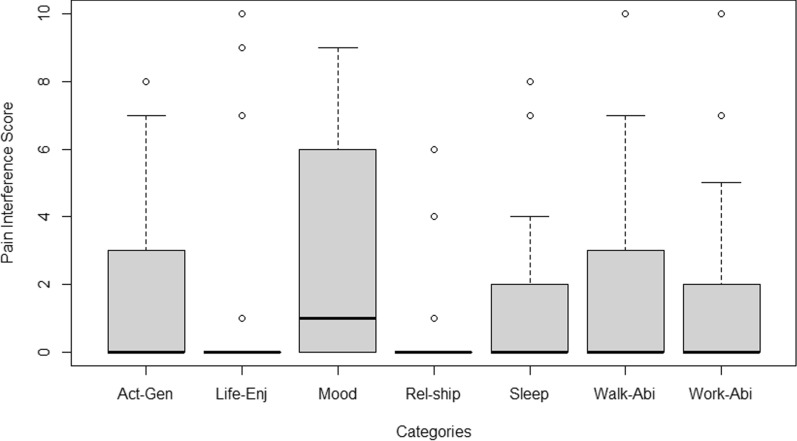
Initial symptoms reported by patients. Source: own elaboration in RStudio®. Act-Gen: activities in general; Life-Enj: enjoy life; Rel-ship: relationships with other people; Walk-Abi: walking ability, Work-Abi: usual work

A median duration of six years was observed after calculating the symptoms for the patients. The IQR spanned from 4 to 14 years, indicating a range of symptom duration. The minimum reported duration was two years, while the maximum reached 30 years. Patients most frequently reported initial symptoms included respiratory distress, limb abnormalities, arm and leg pain, and walking difficulties (Fig. 4).

Patients exhibited growth and development issues, including short stature, weight challenges, and gait delays. Limb bowing was noted by one-third of patients, and roughly 28% experienced activity-limiting bone pain. Tooth problems were common, with 61.1% reporting premature tooth loss and 44.4% permanent tooth loss.

Respiratory history showed 61.1% without previous issues, but 38.9% reported dyspnea, and 16.7% had a history of pneumonia. Muscle concerns included 22.2% with muscle pain and 11.1% with muscle weakness, while most (77.8%) had no muscle-related history. 77.8% reported no renal issues for renal and other medical history, and 83.3% had no other medical history. However, 22.2% had a history of kidney stones, 5.6% had nephrocalcinosis, and 11.1% had elevated blood calcium levels. Additionally, 16.7% showed elevated blood phosphorus levels, and none reported gout history (Table 3).

**Table 3:** Medical history of patients with HPP

	Described conditions	N	%
Growth and development	Short stature (short for age and gender; in case of adults: female < 148.6 cm tall; male < 160.2 cm1)	14	77.8%
	Difficulty gaining weight (feeding difficulties during infancy or childhood)	13	72.2%
	Delayed walking (first walked at 15 months of age or later)	11	61.1%
	Language delay (Delayed in starting to speak)	6	33.3%
	Seizures	4	22.2%
Bone	Bowing of the legs (rickets in the legs)	6	33.3%
	Arching of the arms (rickets in the arms)	6	33.3%
	Genu valgus (knees touching but ankles not touching when standing)	6	33.3%
	Abnormally shaped chest (rib cage abnormalities)	5	27.8%
	Bone pain severe enough to force you to limit your activities	5	27.8%
	Bone pain severe enough to require painkillers	5	27.8%
	Abnormally shaped head (skull)	4	22.2%
	Pseudo-fractures (incomplete fractures or fissures)	4	22.2%
	Unusual gait or walking/running	4	22.2%
	Non-vertebral fracture (broken bone anywhere other than the back)	2	11.1%
	Club foot/Equine foot deformity	2	11.1%
	Vertebral fracture (broken bone in the back)	1	5.6%
	Fractures that don't heal	1	5.6%
Joints	No previous history	9	50.0%
	Joint pain (neck, shoulder, elbow, wrist, wrist, hips, knees, ankles)	7	38.9%
	Extremely flexible joints (hypermobility)	4	22.2%
	Joint inflammation	2	11.1%
Respiratory	Is it serious enough to force you to limit your activities?	3	16.7%
	Is it serious enough to require painkillers?	4	22.2%
	Breathing difficulties	7	38.9%
	Pneumonia	3	16.7%
	non	11	61.1%
Dental	Premature tooth loss (loss of first baby tooth before five years of age)	11	61.1%
	Loss of permanent teeth	8	44.4%
	None	5	27.8%
	Difficulty eating/swallowing	4	22.2%
	Excessive caries	2	11.1%
	Tooth abscess	1	5.6%
Muscles	Muscle weakness	2	11.1%
	Muscle pain	4	22.2%
	none	14	77.8%
Renal	Urolithiasis	4	22.2%
	Nephrocalcinosis (calcium deposits in the kidneys)	1	5.6%
	None	14	77.8%
Other	High levels of calcium in the blood	2	11.1%
	High phosphorus levels in the blood	3	16.7%
	Gout	0	0.0%
	None	15	83.3%

Source: Own elaboration

Fracture incidence analysis in the patient sample revealed that three individuals (16.7%) had experienced bone fractures, all reporting their initial fracture during adolescence. One patient had two fractures (both femur), while two had three fractures each (one involving dorsal vertebra, radius, and carpal bones, and the other involving skull, tibia, and fibula). Multiple fractures resulted from trauma. Additionally, one patient experienced a pseudo fracture, which was promptly diagnosed and took about two to three months to heal.

Surgical history related to hypophosphatasia complications: 44.4% of patients had undergone surgical procedures. Among them, 22.2% mentioned osteotomies, two had kidney stone removal surgeries, one underwent tendon lengthening in both feet, and another had gum surgery for dental obstruction.

In relation to health services, 16.7% received physical therapy and nutritional consultation, while 11.1% received occupational and massage therapy. None of the patients reported receiving respiratory therapy, home health care, or acupuncture. 72.2% of patients did not receive any of the surveyed services. Only 22.2% (four patients) made changes for home modifications due to the disease, primarily in the kitchen, bedroom, and bathroom. Half of the patients did not use any surveyed aids. The most reported aids were orthoses (27.8%), ramps for people with disabilities (22.2%), and manual wheelchairs (22.2%). One patient used respiratory aids, specifically inhalers, for asthma.

In the evolution of hypophosphatasia over the last five years, 72.2% of patients report that it has improved, 22.2% report no change, and only one patient (5.6%) reports that it has worsened. On the other hand, when asked about the symptoms or complications that significantly interfere with their daily lives, ten (55.6%) mentioned pain. However, it is essential to note that the intensity of the reported pain varied among patients.

## Discussion

The clinical manifestations of patients with hypophosphatasia vary from pre- and perinatal mortality without bone mineralization to patients with exclusively dental involvement [16]. Its genotype is as variable as its clinical manifestation, which makes establishing a diagnosis and a clear correlation between genotype and phenotype challenging [17]. In our cohort analysis, we observed patients aged between 3 and 33 years, mainly males. All cases were identified as pediatric-onset hypophosphatasia and were confirmed with molecular studies. Our cohort found ten mutations; one was a mutation for duplication, and the other ones were missense. Two patients had more than one mutation. The most frequently observed genotype was c.892G > A, p.(Glu298Lys), reported in 4 patients, followed by c.382G > A, p.(Val128Met), c.571G > A, p.(Glu191Lys), and c.659G > C, p.(Gly220Ala), each present in 3 patients. Additionally, c.455G > A, p.(Arg152His) was identified in 2 patients. The remaining mutations were found in one patient each.

Eleven variants of the *ALPL* gene were consulted in the databases, and their classification was reviewed. For this purpose, the *ALPL* mutation database was consulted on the 10 of November of 2023 (https://alplmutationdatabase.jku.at/deletions/table/). A total of 438 variants and 733 genotypes have been reported to date. Of the 11 variants documented in our patients, 10 of these have been reported in this database: 9 have been classified as pathogenic, and one (Arg152His) as a variant of uncertain significance (VUS); the latter appears in Clinvar classified as benign or probably benign. The remaining variant (Gly162Asp) does not appear explicitly, but a variant change at the same position is recorded by a different amino acid (Gly162Val), which is classified as pathogenic. This could support pathogenicity of Gly162Asp (under PM5 criteria of the American College of Medical Genetics and Genomics—ACMG).

When comparing our findings to the Michaelus et al. results, which also included 16 patients diagnosed by genetic testing (all confirmed mutations in the *ALPL* gene), the spectrum of their disease is broader, including patients from perinatal HPP to adult-onset cases [18]. Of the symptoms mentioned in the Michaelus study, 68.75% of the patients were noted to have permanent dentition loss, 62.5% exhibited bone deformities, and 50% experienced fractures. In contrast, our patients displayed a lower frequency of fractures and tooth loss, at 16.7% and 44.4%, respectively. Notably, the prevalence of nephrocalcinosis was higher in the Michaelus study (25%) compared to our findings (5.6%). This divergence could be attributed to the higher number of perinatal and infantile HPP cases (4 cases) in our study, where the severity of symptoms is increased.

Additionally, the results obtained in the SF-36 assessment resemble the ones obtained in a recent German study that included 14 adult patients with pediatric-onset hypophosphatasia who underwent ERT for a minimum of 12 months. Although our study did not aim to analyze ERT directly, all our patients received enzyme replacement therapy at some point, which could significantly impact the disease's natural course. In this study, after treatment, the average score for the physical component substantially increased, rising from 26 to 33, while the mental component score improved from 53 to 56. Notably, 13 out of the 14 patients had a history of pain, with nine patients reporting persistent pain at the commencement of treatment. The number decreased to 3 out of 12 patients after six months of treatment. Similarly, our SF-36 questionnaire results highlighted pain as a major complaint. Additionally, patients faced significant challenges with general health and vitality, with some experiencing notable difficulties in role limitations due to physical health and emotional problems. However, most patients maintained an acceptable level of functioning, mental health, and pain control, consistent with findings from a German study following the conclusion of the treatment. [19].

Another study exploring the relationship between pain and quality of life in hypophosphatasia patients showed that those experiencing bone pain have a diminished quality of life compared to those without pain [20]. Our SF-36 questionnaire results revealed that the impact on the quality of life for most patients was also mild to moderate (with a median score above 67%). These findings highlight the correlation between pain and quality of life impairment, as reported by Dahir et al. [20], and align with other studies underscoring the significant impact of pain on quality of life, as Santurtún et al., and Rockman-Greenberg et al. among others [5, 20–23].

Furthermore, among the subset of patients our cohort who disclosed musculoskeletal discomfort, 22% specifically mentioned muscle pain, while 27.8% experienced bone pain of such intensity that it required them to restrict their regular activities. These findings, derived from the HIPS questionnaire, revealed a comparatively lower incidence of pain interference in daily activities. In contrast to our results, other studies have reported proportions of pain nearing 50%, such as Vogt et al. [24], who documented a 48% prevalence of musculoskeletal pain. Noteworthy, Michalus et al. [18] reported chronic bone pain in 56.3% of the patients, and Dahir et al. [25] reported chronic bone pain in 52.5% of the patients with adult-onset HPP.

Another study by Rush et al. conducted an evaluation in patients under 18 years of age with HPP using the HIPS and HOST (HPP Outcomes Study Telephone Interview) surveys [5]. The results revealed that patients encounter a significant disease burden and a decline in their quality of life. Furthermore, it suggests that the disease burden might intensify, and quality of life might deteriorate over time as the symptoms worsen or new symptoms arise. Over five years, around two-thirds of the patients noted a worsening of at least one of their additional signs or symptoms associated with HPP. When comparing to our results, our cohort showed a positive perception of health among the patients, with the majority rating their health as good and a notable percentage considering it excellent. No pain was reported in 50% of the patients. Moreover, participants reported minimal limitations in various physical activities, demonstrating a commendable level of functional ability [5].

The limitations we found in this study are: recall bias must be considered, as patients with more severe symptoms might better recall the disease's onset. Additionally, it is essential to mention that it was decided to use the date of molecular study due to the limitations of the information on the date of clinical diagnosis, given that this information is collected by the patients and not in the medical records, and is therefore influenced by the memory bias that may occur in some of the patients' guardians. This difference in age at diagnosis reported by the caregivers and age at molecular testing was significant in 6 of the 18 patients analyzed. The diagnosis was probably based only on these patients' clinical and paraclinical criteria.

Other limitation is both the SF-36 and BPI scales are validated for adults. However, core aspects can be applicable to children as well considering that lack of existing scales specifically designed for children that cover the same dimensions of quality of life as the adult scale, justifying the need to adapt an adult scale in the study, ensuring maintain the scale’s integrity. We acknowledge that this decision could have generated a bias in the results, but our sample size was limited, and the use of multiple scales may have confounded our analysis. Similarly, we acknowledge that there are more age-appropriate pediatric pain assessment scales. However, to ensure consistency and clarity, we chose a brief and simple scale that is appropriate for a broader age range. Importantly, most of the patients were 8 years old or older (72.2%) and were deemed capable of self-reporting by our psychologist and before using these scales. We recognize that the use of these instruments in the pediatric population has been less frequent [26–28]; however, there is growing evidence to support their validity and reliability for assessing quality of life and pain in children and adolescents [29, 30]. Although there are studies that support the use of these scales in this population, it is important to recognize that their validation and reliability are still limited, which affects the interpretation and comparison of results. Despite these limitations, we believe that our study provides valuable information about quality of life in this population. Therefore, we call for further research in this area, using instruments with robust methodologies that allow us to delve deeper into this aspect.

## Conclusions

The Colombian HPP patient cohort exhibits significant variability in the quality of life, pain, and impact on daily activities. All the patients in our cohort have received ERT. While patients report experiencing pain and reduced quality of life, most describe moderate or mild impairment and interference in their daily routines. According to the validated Spanish version of the HIPS questionnaire, patients generally perceive their overall health as favorable, with most reporting no limitations. Analysis of pain reports indicates consistent responses, with patients either reporting the absence of pain or experiencing mild to moderate intensity.

Regarding medical history, evaluated patients' most frequently observed symptoms and conditions are bone alterations, leading to growth abnormalities, gait problems, dental complications, pain, and respiratory difficulties. These findings align with the natural progression of the disease.

## Data Availability

Data supporting the findings of this study are not openly available due to reasons of sensitivity and to protect the privacy of individuals; however, data are available from the corresponding author upon reasonable request. Data are located in controlled access data storage at Asociación Colombiana de Pacientes con Enfermedades de Depósito Lisosomal (ACOPEL).

## Abbreviations

ACOPEL: Asociación Colombiana de Pacientes con Enfermedades de Depósito Lisosomal
ACMG: American College of Medical Genetics and Genomics
ACMGen: Asociación Colombiana de Médicos Genetistas
BPQ: Brief Pain Questionnaire
ERT: Enzyme replacement therapy
HIPS: Hypophosphatasia Impact Patient Survey
HPP: Hypophosphatasia
HOST: HPP Outcomes Study Telephone Interview
IQR: Interquartile range
NGS: Next Generation Sequencing
SD: Standard deviation
